# Nature of the copper-nitrosyl intermediates of copper nitrite reductases during catalysis†

**DOI:** 10.1039/d0sc04797j

**Published:** 2020-10-20

**Authors:** Michael A. Hough, Jeanet Conradie, Richard W. Strange, Svetlana V. Antonyuk, Robert R. Eady, Abhik Ghosh, S. Samar Hasnain

**Affiliations:** School of Life Sciences, University of Essex Wivenhoe Park Colchester CO4 3SQW UK; Department of Chemistry, Faculty of Natural and Agricultural Sciences, University of the Free State PO Box 339 Bloemfontein South Africa; Department of Chemistry, UiT, The Arctic University of Tromsø 9037 Tromsø Norway; Molecular Biophysics Group, Institute of Systems, Molecular and Integrative Biology, Faculty of Health and Life Sciences, University of Liverpool Liverpool L69 7ZB UK s.s.hasnain@liverpool.ac.uk

## Abstract

The design and synthesis of copper complexes that can reduce nitrite to NO has attracted considerable interest. They have been guided by the structural information on the catalytic Cu centre of the widespread enzymes Cu nitrite reductases but the chemically novel side-on binding of NO observed in all crystallographic studies of these enzymes has been questioned in terms of its functional relevance. We show conversion of NO_2_^−^ to NO in the crystal maintained at 170 K and present ‘molecular movies’ defining events during enzyme turnover including the formation of side-on Cu-NO intermediate. DFT modelling suggests that both *true* {CuNO}^11^ and *formal* {CuNO}^10^ states may occur as side-on forms in an enzymatic active site with the stability of the {CuNO}^10^ side-on form governed by the protonation state of the histidine ligands. Formation of a copper-nitrosyl intermediate thus needs to be accommodated in future design templates for functional synthetic Cu-NiR complexes.

## Abstract 2

Observation of side-on copper-nitrosyl intermediate and its confirmation by DFT during catalysis of copper nitrite reductases.

## Graphical abstract

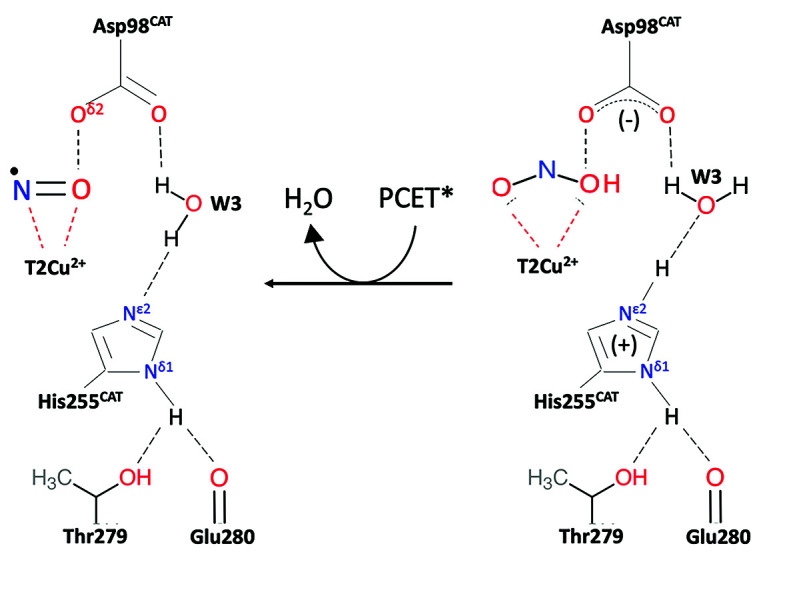

## Introduction

**Scheme 1:**
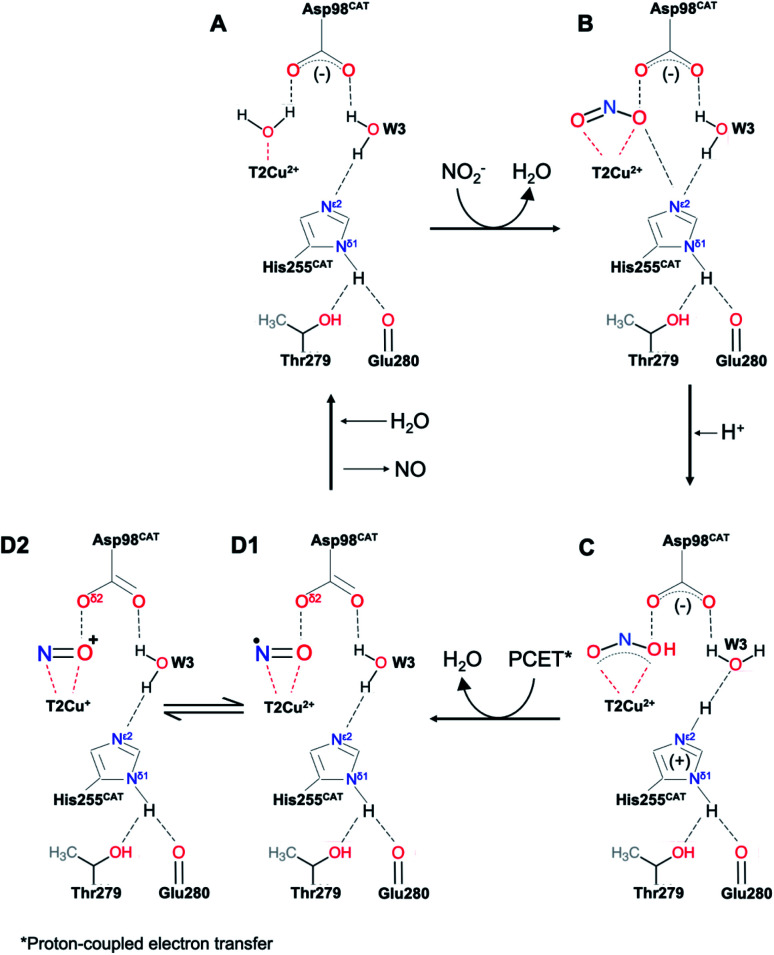
The above scheme incorporates the salient features of recent structures of *Ac*NiR (and those shown here) determined using synchrotron radiation, neutron diffraction and X-ray free electron lasers. In the resting state (A) both His_CAT_ and Asp_CAT_ are deprotonated, water bound to T2Cu is displaced by nitrite which binds *via* two oxygen atoms, (B), prompting a proton uptake from the bulk solvent by Asp_CAT_. The subsequent proton transfer to nitrite leads to the protonated nitrite intermediate in (C). Following the protonation of His_CAT_ from bulk solvent a transfer of a proton from the bridging water (W3) and electron transfer from T1Cu to T2Cu-nitrite intermediate results in NO bond cleavage, forming H_2_O and the nitrosyl product in D1–D2. This scheme differs from numerous earlier proposals in that the PCET reaction that controls the rate of turnover is associated with the transfer of the second proton in NiR turnover.

Copper nitrite reductases (CuNiRs) found in fungi, bacteria and archaea belong to a highly conserved enzyme family that catalyze the one-electron reduction of nitrite to NO [NO_2_^−^ + e^−^ + 2H^+^ ⇆ NO + H_2_O]. The involvement of CuNiRs in two branches of the global nitrogen cycle, denitrification^1^ and nitrification,^2^ make them of critical environmental importance since N_2_O is a major by-product of denitrification, which is the third most significant greenhouse gas with a global warming potential 300 times that of CO_2_.^3^ The catalytic type 2 Cu centre (T2Cu) of CuNiR is located at the interface of two adjacent monomers and linked to the electron-donating T1Cu centre *via* a Cys–His bridge. Two invariant active site pocket residues Asp_CAT_ and His_CAT_ are involved in substrate-binding and catalysis and a conserved Ile_CAT_ residue provides steric control of ligand binding to the T2Cu. The unexpected involvement of side-on *vs.* end-on NO binding during crystal soaking and also in the CuNiR catalytic cycle revealed in numerous structural studies has attracted much attention, with several proposals as to its origin. It has been suggested that the side-on NO binding mode may be ‘frozen out’, as crystal structures determined until 2007 had all been obtained from crystals maintained at 100 K and may be caused by interactions of bound NO with the type 2 Cu pocket residues Ile_CAT_ ^4^ and Asp_CAT_.^5^ This issue has recently been highlighted in biomimetic studies,^6^ where the characterisation of a {CuNO}^10^ electron-configuration species with end-on binding of NO formed either by reduction of nitrite at, or NO-soaking into, Cu(i) dichloride or its bromide analogue has been described. In each case, X-ray crystallography of the synthetic system revealed an end-on Cu–N–O binding mode. These observations in model systems may lead to the erroneous conclusion that the side-on NO binding observed in crystal structures of CuNiR enzymes^7,8^ is the isolation of a frozen-out one-electron reduced {CuNO}^11^ species that may not be relevant to the enzymes' catalytic cycle. A recent 100 K neutron crystallographic study of the resting state of CuNiR from *Geobacillus thermodenitrificans*, has suggested that the catalytic T2Cu is coordinated by a hydroxide ion and not water. Despite the presence of some adventitious copper bound to one of the catalytic residues, the coordination of hydroxide has been taken to suggest that Cu^II^–OH^−^ is the stable intermediate after reduction of nitrite in a mechanism not involving the formation of a Cu-nitrosyl adduct prior to NO release.^9,10^ This puts the observation of the Cu-nitrosyl adduct in crystal structures of several CuNiRs in question, Scheme 1. Only recently a simple copper motif was described that could mediate the evolution of NO^−^ from nitrite with evidence of a {CuNO}^10^ species adding chemical evidence for a {CuNO}^10^ intermediate in the mechanism of CuNiR enzymes.^6^

We have undertaken crystallographic studies at a higher temperature of 170 K (normal temperature for synchrotron X-ray data collection is 100 K) at atomic resolution (<1.20 Å) confirming the formation of a side on nitrosyl intermediate during catalysis in crystals and show by DFT calculations that the stability of the {CuNO}^10^ side-on form is governed by the protonation state of the histidine ligands in the catalytic pocket. For {CuNO}^11^, both end-on and side-on forms are energetically feasible with the occurrence of a side-on {CuNO}^11^ intermediate in an enzymatic active site, but energetically unfavourable for its the isolation in a synthetic complex, except at low temperature.

## Results

### *In situ* formation of Cu-NO in crystals of nitrite-soaked copper nitrite reductases

For enzymes that use chemical substrates, structural information on potential reaction intermediates has been largely limited to static structures obtained by soaking diffracting crystals with substrate, inhibitors and their analogues. In the case of nitrite reduction by CuNiRs, an electron is a substrate in addition to the chemical substrate nitrite. An opportunity is thus provided to use the electrons generated within the crystal by exposure to X-rays to trigger the catalytic conversion of the chemical substrate *in situ*. It has been previously established that electrons generated during X-ray radiolysis are transferred to the catalytic site *via* the type-1 Cu site that is also utilized by physiological electron donors.^11–13^ The electron delivery from T1Cu to the catalytic T2Cu is gated in the same manner as the physiological or artificial electron donor *i.e.* under these conditions, electrons are transferred from T1Cu to T2Cu only in the presence of nitrite.

By controlling the X-ray dose and using efficient and fast detectors, a large number of structures can be obtained from the same volume of a crystal. This allows visualisation of the conversion of the substrate, nitrite to nitric oxide and capture the performance of the catalytic pocket orchestrating the conversion and subsequent release of NO before returning to the resting state.^14,15^ This approach to serially obtain multiple structures from a single CuNiR crystal (MSOX) has been used here to provide a new atomic-resolution series with crystal maintained at 170 K. In this series, we have focussed on the generation of the product and the initial steps of its release, documenting bond breakage and the act of product formation at atomic resolution (<1.20 Å resolution), Table S1.† The final data set (5^th^ in the series), where the enzyme has partially returned to the resting state after releasing the product, has been defined at 1.15 Å with the Cu coordinated to a mixture of water and NO.

**Fig. 1:**
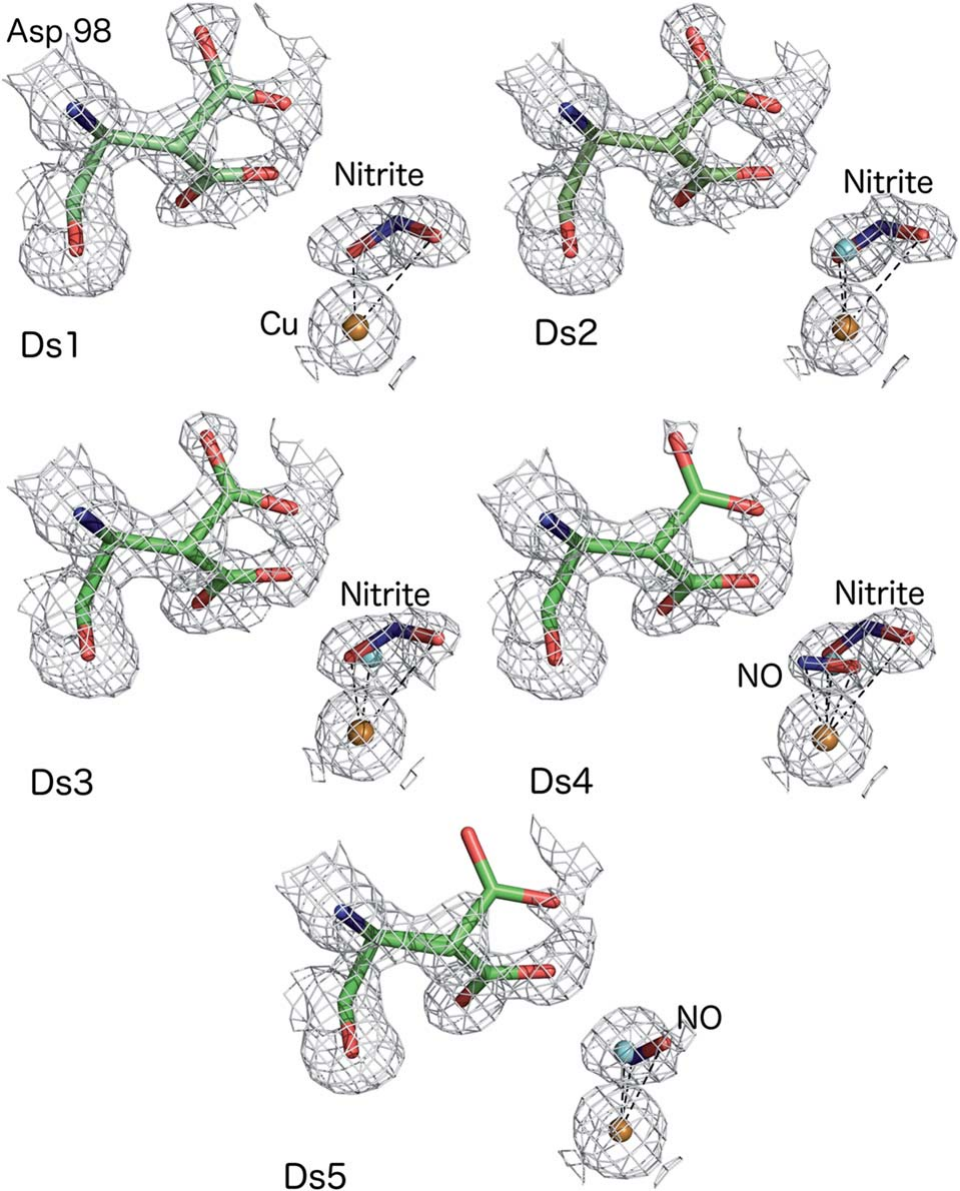
MSOX series of nitrite-soaked *Ac*NiR crystal at 170 K showing bidentate binding of nitrite (Ds1–3), conversion of nitrite to side-on NO with nitrite present at around 0.4 occupancy (Ds4), side-on NO with disappearance of nitrite and some T2Cu sites returning to the resting state with water bound form (Ds5). Water molecules present in Ds2–5 are shown as cyan spheres for clarity.

The atomic resolution of these crystallographic data sets allowed us to deploy SHELXL for an unrestrained refinement and determine the structural details with sufficient accuracy to underpin the chemistry of the catalytic site during turnover. SHELXL has provided occupancy refinement for ligands as well as multiple conformations of Asp_CAT_ and provide uncertainties for bond lengths and angles (calculated uncertainties are included in the brackets and correspond to the last digit(s) of the distances/angles). In dataset 1 (Ds1), clear electron density is apparent for a nitrite molecule bound to the catalytic type 2 Cu site in a highly asymmetric bidentate orientation (Fig. 1) with Cu–O_2_ and Cu–O_1_ (nitrite) bonds 1.88(3) and 2.51 Å, respectively with an occupancy of 0.75 nitrite. The Cu–N (nitrite) distance was 2.42(6) Å. Dataset 4 shows the clear emergence of the side-on NO species, bound to Cu by O at 1.65(1) and N at 1.86(2) Å with an occupancy of 0.3 and nitrite coordinated to Cu by O_2_ atom at 2.09(10) Å with the O1 atom now at 2.70 Å, with nitrite still present at an occupancy of 0.4. The NO occupancy increases to 0.35 in dataset 5, where it remains in a side-on geometry but beginning to show a tilt (the distance Cu–O is now 2.02(10) Å and Cu–N is 1.86(13)). Asp98_CAT_ is now almost fully (0.82) in the proximal orientation, with a small fraction in the gatekeeper position.^16^ In datasets 2–5 a partial occupancy water molecule is modelled co-ordinated to the T2Cu.

### DFT calculations on the occurrence of side-on CuNO intermediates in CuNiR

Because DFT calculations on the full catalytic pathway have so far failed to shed light on the side-on CuNO intermediate species,^10,17^ we chose to focus here specifically on the stability and electronic configuration of such species, a question that DFT calculations have successfully addressed for a variety of nitrosyl systems.^18–23^ Toward that end, the ligand environment of the *Ac*NiR enzyme was modelled by either the monoanionic hydrotris(4-imidazolyl)borate (L1) ligand or a dianionic ligand (L2) consisting of L1 with an acetate side-chain at C2 of the imidazole rings hydrogen-bonding with the imidazole NH. Both ligands are inspired by the scorpionates, especially hydrotris(pyrazolyl)borate and its substituted analogues, which have been widely used to model nonheme active sites.^24,25^ With these ligands, the optimized geometries of the complexes of interest all conformed closely to *C*_s_ symmetry and accordingly are reported as such.

**Fig. 2:**
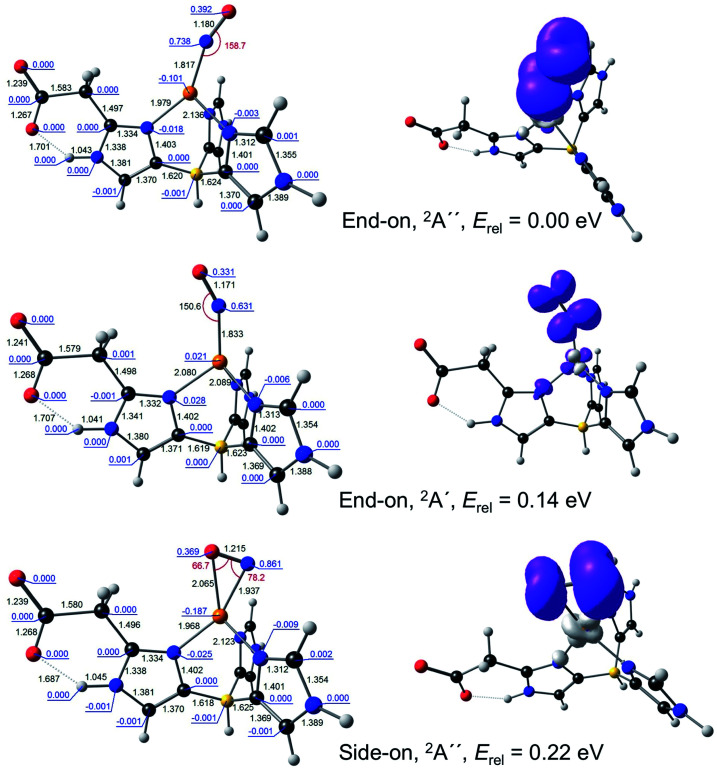
Selected B3LYP/STO-TZ2P results for {CuNO}^11^–L2. Left: Optimized distances (black, Å), angles (red, °), and Mulliken spin populations (blue). Right: Spin density plots.

For {CuNO}^11^, both end-on and side-on forms could be readily optimized (Fig. 2). The end-on form was actually obtained as one of two nearly equi-energetic symmetry states, ^2^A′ and ^2^A′′, whereas the side-on form was unambiguously assigned to a ^2^A′′ state (using the group-theoretic notations for the irreducible representations for the *C*_s_ point group). The unpaired electron in all these states was found to occupy an NO π* orbital. For all these states, the Cu–N distance was well under 2.0 Å, reflecting the importance of Cu-NO back-bonding, with the end-on form exhibiting slightly shorter Cu–N and NO distances relative to the side-on form. For both L1 and L2, the energy of the side-on form was about a quarter-eV higher than that of the lowest-energy end-on form. This margin of energy is small enough to be readily compatible with the occurrence of a side-on {CuNO}^11^ intermediate in the enzymatic active site, but large enough to discourage the isolation of such a form for a synthetic complex, except at low temperature.

**Fig. 3:**
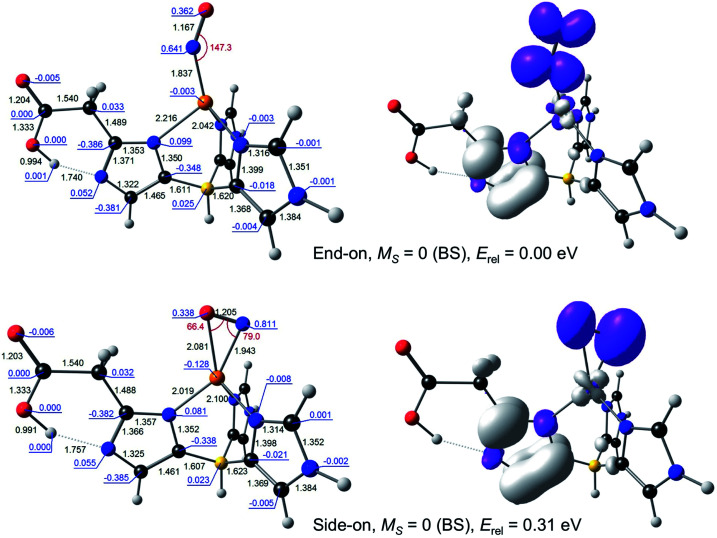
Selected B3LYP/STO-TZ2P results for broken-symmetry *M*_S_ = 0 solutions for {CuNO}^10^–L2. Left: Optimized distances (black, Å), angles (red, °), and Mulliken spin populations (blue). Right: Spin density plots.

For {CuNO}^10^, a broken-symmetry *M*_S_ = 0 side-on form proved impossible to converge with L1 as the supporting ligand. An *M*_S_ = 1 side-on form could be converged, albeit at 0.66 eV relative to the *M*_S_ = 0 end-on form, a rather high energy even for an enzyme active site. In contrast, both *M*_S_ = 0 and *M*_S_ = 1 side-on forms could be readily located with L2 as the supporting ligand, at an energy of about 0.3 eV, relative to the ground state (Fig. 3). Intrigued by the low energy of these side-on species, we carefully scrutinised their Kohn–Sham MOs and spin density profiles. It quickly became apparent that with L2 as the supporting ligand, the putative {CuNO}^10^ states, end-on or side-on, are actually {CuNO}^11^ species coupled to a radical derived from oxidation of the acetate-bearing imidazole. Such a description parallels that for formal {FeNO}^6^ corroles, which are now thought to be better described as {FeNO}^7^-corrole˙^2−^ assemblies.^26,27^ This insight also explains the surprisingly similar geometries of the CuNO units for the {CuNO}^10^ and {CuNO}^11^ states for the L2 supporting ligand.

The calculations thus indicate that both *true* {CuNO}^11^ and *formal* {CuNO}^10^ states may occur as side-on forms in an enzymatic active site. The stability of the {CuNO}^10^ side-on form, however, should depend a great deal on the exact protonation state of the histidine ligands. An electron-rich histidinate-type ligand will go a long way toward stabilizing such a state by harbouring the oxidizing equivalent as a radical.

## Discussion

The MSOX approach we have used provides direct visualisation of what happens in the whole of the catalytic pocket during the reduction of nitrite (Fig. 1). The fact that the reaction can be followed structurally in the same crystal, from nitrite-bound to side-on NO intermediate prior to returning to the resting state, shows that side-on NO is the enzyme-bound intermediate of the catalytic cycle defining the product formation event. The MSOX experiments described here provide structures of species generated during catalysis in a single turnover event triggered by the generation of reductant. The identification of an Cu nitrosyl intermediate is consistent with solution studies of CuNiR where EPR characterization identified a Cu(i)NO species generated in single turnover of *Rs*NiR under reductant limited conditions^28^ and showed hyperfine features associated with both Cu and ^14^NO. Analysis of the EPR data, together with complementary ENDOR studies was consistent with spin delocalisation over the Cu and N of NO and a dipolar interaction of the spin of NO and the C^δ1^ protons of Ile_CAT_.

The MSOX series at 170 K, reported here, at atomic resolution is comparable with those determined at other temperatures,^10^ reproducing key mechanistic features including the orientation of nitrite and its bidentate mode of binding, changes to occupancy of the gatekeeper and proximal rotamer conformations of Asp_CAT_ and the formation of an NO intermediate, demonstrating the presence of side-on NO in crystals of CuNiR during an *in situ* turnover. The characterization of a stable nitrosyl intermediate with side-on binding in multiple crystal structures and MSOX experiments provides compelling evidence that such a side on intermediate is indeed formed during catalysis (Movies S1 and S2†).

**Fig. 4:**
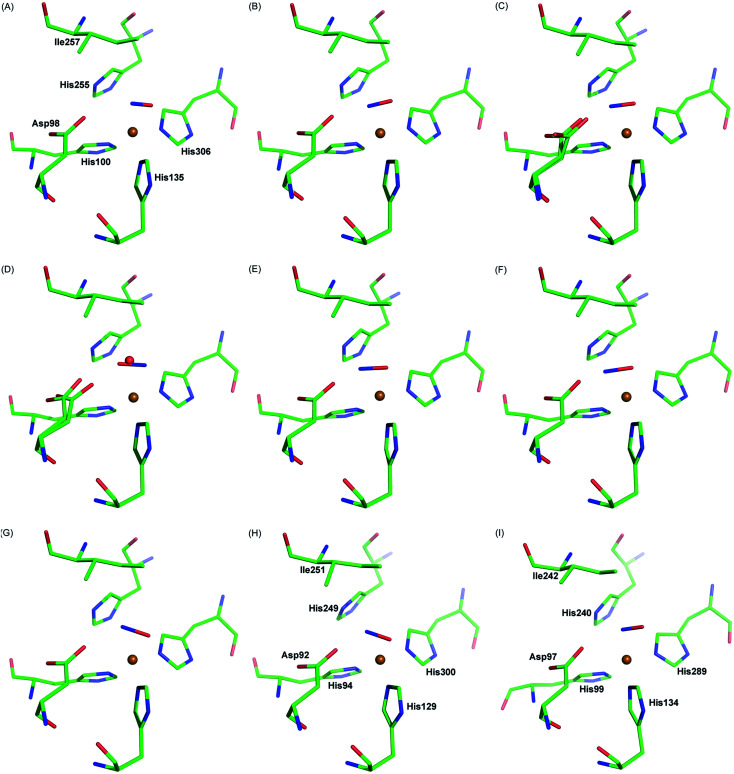
Side-on NO-binding observed at the T2Cu site at different temperatures and in different CuNiRs. Panels A–C: MSOX frames of *Ac*NiR showing NO generated *in situ* by radiolysis at room temperature (A; PDB 5og4), 190 K (B; PDB 5of8) and 100 K (C; PDB 5i6n). Panel D: *Ac*NiR at 100 K with endogenous NO (PDB 2bw5). Panels E–G: *Af*NiR (E; PDB 1snr) and the oxidised (F; PDB 2ppd) and reduced (G; PDB 2ppe) *Af*NiR H145A mutant exposed to NO. Panel H: *Ax*NiR (PDB 2xwz). Panel I: the three domain *Rp*NiR (PBD 5ocf).

Our observation is consistent with previous single crystal structures of NO-bound *Ac*NiR^16^ and other CuNiRs,^7,8,29^Fig. 4. Similar side-on binding has been observed in NO soaked crystals for three domain heme-CuNiR from *Ralstonia pickettii* (*Rp*NiR) and its Asp_CAT_ mutant^30^ and the three domain cupredoxin-CuNiR from *Hyphomicrobium denitrificans* strain 1NES1 (Hd_1NES1_NiR),^31^ demonstrating the universality of this species in all classes of copper nitrite reductases. Furthermore, the presence of the side-on binding mode of NO at multiple temperatures suggests that this is not an artefact caused by cryo-cooling of the crystalline enzyme (Fig. 4, Movies S1 and S2†). The active site pocket residue Asp 98 changes its rotamer during the MSOX series in response the status of ligand at the T2Cu site hence indicating the responsive nature of the catalytic pocket in the crystal. These observations are consistent with the catalytic competency of crystalline enzymes in a number of room temperature studies *e.g.*ref. 32, where chemical reactions and electron/proton transfers have been shown to occur. This is not unexpected as protein molecules are immersed in solvent in the crystals in the same manner as they are in solution at high concentrations.

In addition, the reversibility of the reaction catalysed by CuNiRs and the formation of N_2_O during turnover in the presence of NO are consistent with the formation of a Cu-nitrosyl species in solution at ambient temperature. The latter reaction is an example of the combination of an N atom of NO_2_^−^ and an N atom of a co-metabolised compound (*e.g.* NO, NH_3_, HONH_2_) resulting in the formation of hybrid N–N products including N_2_. Isotopic labelling has established that the N atom of nitrite is incorporated into the N–N product. This process, termed co-denitrification, is widespread in biology and is an important contributor to the N-cycle in agriculture.^33,34^ This, taken together with the extensive body of structural data including that which we describe herein, supports the formation of a Cu nitrosyl species, rather than the alternative hypothesis that proton-coupled electron transfer to nitrite-bound T2Cu is followed by reduction of nitrite to NO which is released without forming a Cu-nitrosyl.^9,10,35^

**Scheme 2:**
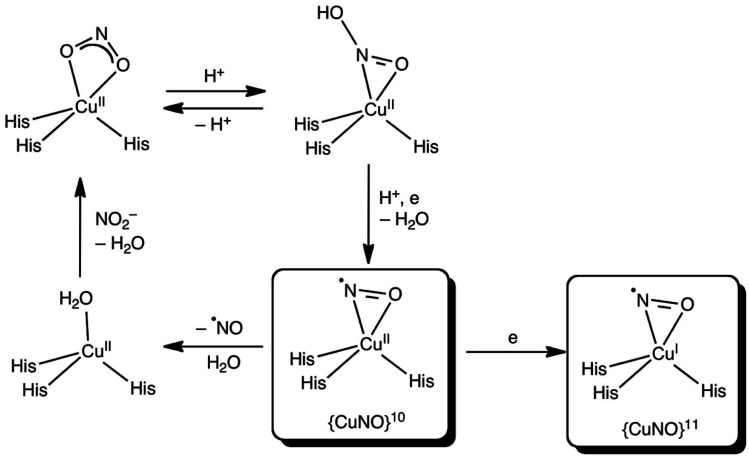
Proposed reaction scheme for the T2 site of CuNiR with side-on {CuNO}^10^ and {CuNO}^11^ species (see above text for details).

Which side-on CuNO intermediate is being observed in the MSOX? This question may be discussed with reference to Scheme 2. Based on the present DFT results, a side-on, formal {CuNO}^10^ species with one or more hydrogen-bonded histidine ligands is a potential candidate. Such a species may be viewed as an antiferromagnetically coupled {CuNO}^11^–His˙ species. Alternatively, such a species may be reduced even further to a true {CuNO}^11^ state, for which side-on coordination is even more favourable. It is important to recognize that the actual reaction mechanism of CuNiRs is expected to be critically dependent on the exact hydrogen-bond and protonation state and dynamics of the active site, which are unlikely to be fully captured in either synthetic models or computational cluster models of the enzyme. Thus, the side-on states observed in the present studies may prove elusive in synthetic and computational modelling studies.^36^

## Materials and methods

Crystallisation of *Ac*NiR enzyme was performed as described previously.^14–16^ Crystals grew in space group *P*2_1_3 and were cryoprotected and flash-cooled as previously described,^14–16^ being mounted in a cryo-loop for X-ray data collection. An MSOX series of 5 datasets was collected at Swiss Light Source beamline X10SA using a Pilatus 6M-F detector and an X-ray wavelength of 0.7 Å. The crystal was maintained in a nitrogen cryostream throughout the experiment. In this approach, the same exposed volume of one *Ac*NiR enzyme crystal was used for measurement of repeated, complete crystallographic datasets. Generation of solvated photoelectrons by the X-ray beam provides the driving force for the enzyme reaction, with movie ‘frames’ produced at increasing dose points. Data were processed using XDS^37^ and then scaled & merged in Scala^38^ within the CCP4 suite. Refinement was performed using Refmac5 ^39^ in the CCP4 suite with rebuilding in Coot.^40^ Datasets were refined using anisotropic B-factors. Nitrite ligands were fitted using the ‘find ligands’ feature of Coot. This was followed by restrained refinement in SHELX-97,^41^ where refinement of anisotropic B-factors, hydrogen positions, occupancies of the double conformation of the side chains and T2Cu ligands was implemented. Refinement parameters are given in Table S1.† At the final stage of the refinement one cycle of unrestrained block-matrix least-squares refinement was implemented to estimate the standard deviations (e.s.d.s) of coordinates and derived parameters (bond lengths and angles). The geometric parameters were refined in cycle 1 followed by the anisotropic displacement parameters refinement. Information on the selected Cu ligands bonding and Asp_CAT_98 bonds with e.s.d.s are shown in Table S2.† Validation was performed using Molprobity,^42^ JCSG QC-check and tools in CCP4. The MSOX movies (190 K and room temperature) were constructed from structures already deposited^10^ in the RCSB Protein Data Bank.^43^ Figures and movies were prepared using PyMol.^44^

All DFT calculations were carried out with the ADF program system,^45^ using the B3LYP^46,47^ exchange–correlation functional (20% Hartree–Fock exchange) and the all-electron Slater-type TZ2P basis sets and full geometry optimization in vacuum. The calculations were allowed to break spin symmetry, wherever relevant. Different electronic configurations were studied *via* a *C*_s_ symmetry constraint and manually specifying electron occupancies for each irreducible representation. The results proved stable across a wide range of exchange–correlation functionals. Note, however, that broken-symmetry solutions for *M*_S_ = 0 systems are often only obtainable with hybrid functionals (such as B3LYP, PBE0, and M06-L),^18–27^ which was also the case in this study.

## Conflicts of interest

Authors have no conflicts to declare.

## Supplementary Material

SC-011-D0SC04797J-s001

SC-011-D0SC04797J-s002

SC-011-D0SC04797J-s003
