# Supplementary material for: Nature of the copper-nitrosyl intermediates of copper nitrite reductases during catalysis
Source: Chem Sci. 2020 Oct 20;11(46):12485–92. doi: 10.1039/d0sc04797j (PMC8163067; doi:10.1039/d0sc04797j)
Supplement: SC-011-D0SC04797J-s002 [file SC-011-D0SC04797J-s002.pdf]

Electronic Supplementary Material (ESI) for Chemical Science.
This journal is © The Royal Society of Chemistry 2020

Supporting information

Table of Contents

Nature of the copper-nitrosyl intermediates of copper nitrite reductases during catalysis 1
Table of Contents 1
DFT method 2
1. $\{CuNO\}^{10}$ - L2, End-on, M_S = 0 (BS) 2
2. $\{CuNO\}^{10}$ - L2, Side-on, M_S = 0 (BS) 3
3. $\{CuNO\}^{11}$ - L2, Side-on, $^2A''$ 3
4. $\{CuNO\}^{11}$ - L2, End-on, $^2A''$ 4
5. $\{CuNO\}^{11}$ - L2, End-on, $^2A'$ 5

Optimized Cartesian coordinates (Å)

DFT method

All DFT calculations were carried out with the ADF program system[35], using the B3LYP[36a,b] exchange-correlation functional (20% Hartree-Fock exchange) and the all-electron Slater-type TZ2P basis sets. The calculations were allowed to break spin symmetry, wherever relevant. Different electronic configurations were studied via a C_s symmetry constraint and manually specifying electron occupancies for each irreducible representation.

$\{CuNO\}^{10}$ - L2, End-on, M_s = 0 (BS)

Cu	-0.600699000	0.989951000	0.000000000
B	-0.821163000	-2.102475000	0.000000000
C	0.692489000	-1.561710000	0.000000000
C	1.983791000	-2.245301000	0.000000000
C	2.350546000	-0.149358000	0.000000000
C	3.076253000	1.149238000	0.000000000
C	4.615915000	1.169977000	0.000000000
C	-1.542889000	-1.473127000	1.310397000
C	-1.542889000	-1.473127000	-1.310397000
C	-2.236591000	-2.048056000	2.340585000
C	-2.236591000	-2.048056000	-2.340585000
C	-2.266757000	0.144533000	2.607371000
C	-2.266757000	0.144533000	-2.607371000
H	2.150803000	-3.312017000	0.000000000
H	2.755275000	1.739516000	0.864115000
H	2.755275000	1.739516000	-0.864115000
H	4.619562000	-0.749730000	0.000000000
H	-0.858799000	-3.310267000	0.000000000
H	-2.454570000	-3.077754000	2.560773000
H	-2.454570000	-3.077754000	-2.560773000
H	-2.479476000	1.110703000	3.033701000
H	-2.479476000	1.110703000	-3.033701000
H	-3.233923000	-1.112930000	3.989198000
H	-3.233923000	-1.112930000	-3.989198000
N	0.997232000	-0.244563000	0.000000000
N	2.973242000	-1.364635000	0.000000000
N	-1.403127000	2.759408000	0.000000000
N	-1.576645000	-0.090739000	1.513752000
N	-1.576645000	-0.090739000	-1.513752000
N	-2.690511000	-1.019728000	3.147855000
N	-2.690511000	-1.019728000	-3.147855000
O	5.191640000	2.225990000	0.000000000
O	5.263107000	0.004165000	0.000000000
O	-0.230501000	3.038245000	0.000000000

{CuNO}^{10} - L2, Side-on, M_s = 0 (BS)

Cu	0.660618000	-1.222287000	0.000000000
B	1.408998000	1.815669000	0.000000000
C	1.964083000	1.042767000	1.311559000
C	1.964083000	1.042767000	-1.311559000
C	2.289428000	-0.692943000	2.622378000
C	2.289428000	-0.692943000	-2.622378000
C	2.706405000	1.462426000	2.381636000
C	2.706405000	1.462426000	-2.381636000
C	-0.192717000	1.643428000	0.000000000
C	-1.274423000	2.631196000	0.000000000
C	-2.134512000	0.687692000	0.000000000
C	-3.162323000	-0.389874000	0.000000000
C	-4.658419000	-0.026810000	0.000000000
H	1.728556000	2.982091000	0.000000000
H	2.285869000	-1.682655000	3.046820000
H	2.285869000	-1.682655000	-3.046820000
H	3.108313000	2.428899000	2.628120000
H	3.108313000	2.428899000	-2.628120000
H	3.416101000	0.342618000	4.063953000
H	3.416101000	0.342618000	-4.063953000
H	-1.173307000	3.706500000	0.000000000
H	-2.997886000	-1.040614000	0.864155000
H	-2.997886000	-1.040614000	-0.864155000
H	-4.177583000	1.831499000	0.000000000
N	1.716212000	-0.321276000	1.497735000
N	1.716212000	-0.321276000	-1.497735000
N	2.905948000	0.362320000	3.197297000
N	2.905948000	0.362320000	-3.197297000
N	-0.102565000	-2.893453000	0.000000000
N	-0.804359000	0.440068000	0.000000000
N	-2.448313000	2.022173000	0.000000000
O	0.062491000	-4.049180000	0.000000000
O	-4.992969000	1.263578000	0.000000000
O	-5.481052000	-0.905692000	0.000000000

{CuNO}^{11} - L2, Side-on, ^{2}A″

Cu	-0.561397000	0.993313000	0.000000000
B	-0.794824000	-2.099206000	0.000000000
C	0.733777000	-1.568109000	0.000000000
C	1.947368000	-2.203649000	0.000000000
C	2.333939000	-0.032186000	0.000000000
C	3.121985000	1.239789000	0.000000000
C	4.697803000	1.120366000	0.000000000
C	-1.544723000	-1.484117000	1.303217000
C	-1.544723000	-1.484117000	-1.303217000

C	-2.222285000	-2.053437000	2.348191000
C	-2.222285000	-2.053437000	-2.348191000
C	-2.239740000	0.139669000	2.617308000
C	-2.239740000	0.139669000	-2.617308000
H	2.185455000	-3.252956000	0.000000000
H	2.849566000	1.842783000	0.869899000
H	2.849566000	1.842783000	-0.869899000
H	3.971605000	-1.193652000	0.000000000
H	-0.856489000	-3.314445000	0.000000000
H	-2.429627000	-3.083310000	2.579857000
H	-2.429627000	-3.083310000	-2.579857000
H	-2.437741000	1.107638000	3.048153000
H	-2.437741000	1.107638000	-3.048153000
H	-3.166827000	-1.114033000	4.033169000
H	-3.166827000	-1.114033000	-4.033169000
N	1.009277000	-0.193051000	0.000000000
N	2.927697000	-1.230842000	0.000000000
N	-1.482959000	2.696951000	0.000000000
N	-1.579365000	-0.098750000	1.508972000
N	-1.579365000	-0.098750000	-1.508972000
N	-2.657219000	-1.023235000	3.171996000
N	-2.657219000	-1.023235000	-3.171996000
O	5.204332000	-0.042168000	0.000000000
O	5.285085000	2.211286000	0.000000000
O	-0.318613000	3.043903000	0.000000000

$\{CuNO\}^{11}$ - L2, End-on, $^{2}A''$

Cu	0.520460000	1.043518000	0.000000000
B	1.117182000	-2.008171000	0.000000000
C	1.783204000	-1.314305000	1.308120000
C	1.783204000	-1.314305000	-1.308120000
C	2.265894000	0.368055000	2.642867000
C	2.265894000	0.368055000	-2.642867000
C	2.488907000	-1.812950000	2.370821000
C	2.488907000	-1.812950000	-2.370821000
C	-0.465019000	-1.660301000	0.000000000
C	-1.591301000	-2.440690000	0.000000000
C	-2.239779000	-0.332490000	0.000000000
C	-3.178160000	0.834321000	0.000000000
C	-4.730610000	0.527365000	0.000000000
H	1.317776000	-3.208734000	0.000000000
H	2.346554000	1.348710000	3.082846000
H	2.346554000	1.348710000	-3.082846000
H	2.794761000	-2.816595000	2.608151000
H	2.794761000	-2.816595000	-2.608151000
H	3.283012000	-0.786370000	4.082193000
H	3.283012000	-0.786370000	-4.082193000
H	-1.698430000	-3.511299000	0.000000000
H	-2.981267000	1.465575000	0.870479000

H	-2.981267000	1.465575000	-0.870479000
H	-3.721205000	-1.691223000	0.000000000
N	0.863195000	2.827948000	0.000000000
N	1.665943000	0.066296000	1.515490000
N	1.665943000	0.066296000	-1.515490000
N	2.789603000	-0.746193000	3.207812000
N	2.789603000	-0.746193000	-3.207812000
N	-0.905736000	-0.328694000	0.000000000
N	-2.683061000	-1.595196000	0.000000000
O	1.491074000	3.826601000	0.000000000
O	-5.093450000	-0.686785000	0.000000000
O	-5.443121000	1.541290000	0.000000000

$\{CuNO\}^{11}$ - L2, End-on, $^{2}A'$

Cu	-0.757371000	0.989735000	0.000000000
B	-0.835451000	-2.118764000	0.000000000
C	0.687356000	-1.570348000	0.000000000
C	1.901490000	-2.207963000	0.000000000
C	2.275071000	-0.035795000	0.000000000
C	3.059727000	1.240170000	0.000000000
C	4.634896000	1.136282000	0.000000000
C	-1.587527000	-1.518252000	1.306985000
C	-1.587527000	-1.518252000	-1.306985000
C	-2.188390000	-2.106841000	2.387434000
C	-2.188390000	-2.106841000	-2.387434000
C	-2.285090000	0.084644000	2.645945000
C	-2.285090000	0.084644000	-2.645945000
H	2.140595000	-3.257408000	0.000000000
H	2.783372000	1.842122000	0.869652000
H	2.783372000	1.842122000	-0.869652000
H	3.919168000	-1.198893000	0.000000000
H	-0.883168000	-3.335622000	0.000000000
H	-2.332269000	-3.143092000	2.637708000
H	-2.332269000	-3.143092000	-2.637708000
H	-2.500170000	1.044534000	3.086295000
H	-2.500170000	1.044534000	-3.086295000
H	-3.087444000	-1.196283000	4.112193000
H	-3.087444000	-1.196283000	-4.112193000
N	0.952802000	-0.193863000	0.000000000
N	2.878606000	-1.233265000	0.000000000
N	-0.703005000	2.821911000	0.000000000
N	-1.673219000	-0.133029000	1.504464000
N	-1.673219000	-0.133029000	-1.504464000
N	-2.624642000	-1.090504000	3.226684000
N	-2.624642000	-1.090504000	-3.226684000
O	5.153956000	-0.020391000	0.000000000
O	5.212708000	2.234330000	0.000000000
O	-0.098505000	3.825044000	0.000000000

SUPPLEMENTARY DATA

Movie S1: MSOX movie at 190K movie constructed from 75 structures serially obtained from one crystal.

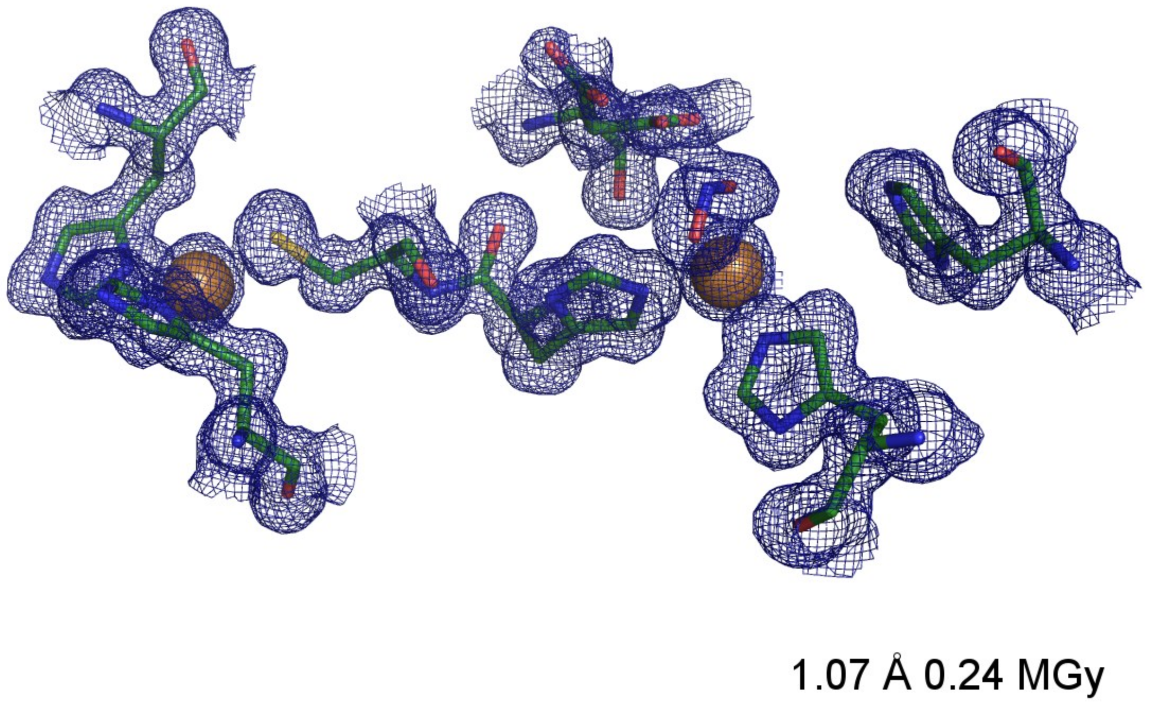

Movie S2: MSOX movie at room temperature constructed from 10 structures serially obtained from one crystal.

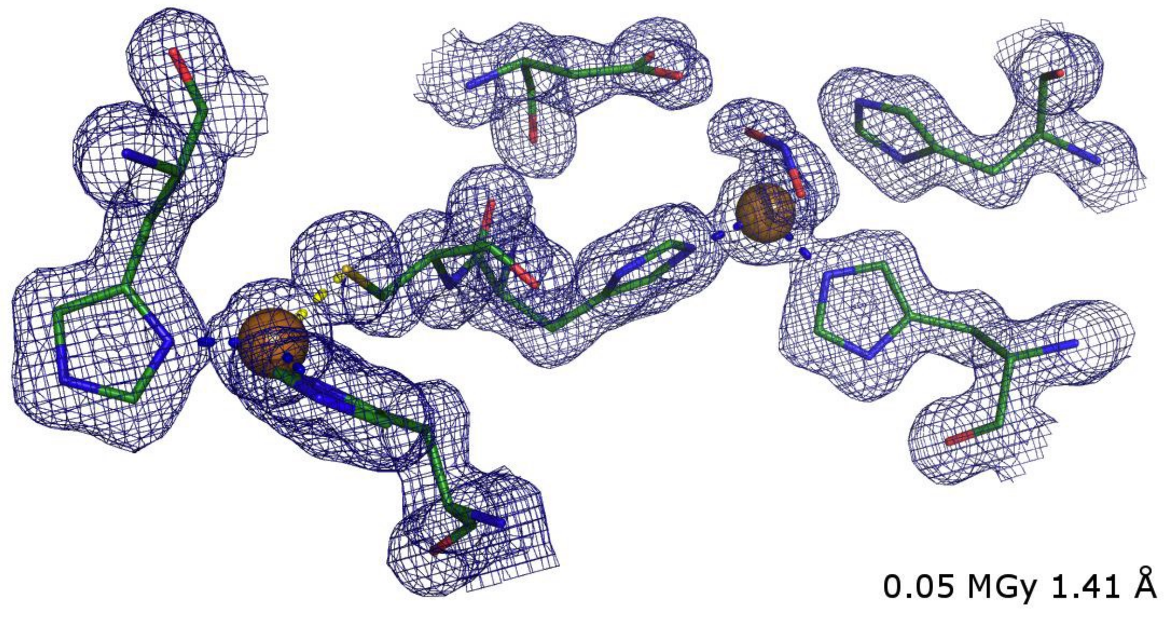

Table S1. MSOX series of nitrite-soaked *Ac*NiR at 170 K

**Structure**	$ds1_{170}$	$ds2_{170}$	$ds3_{170}$	$ds4_{170}$	$ds5_{170}$
Cell axis (Å)	95.3	95.4	95.4	95.4	95.4
Resolution (Å)	42.6-1.15	42.6-1.08	42.6-1.10	42.6-1.14	42.6-1.15
Unique reflections	101516	122039	115774	104229	101537
Redundancy	5.1 (5.1)	4.8 (3.5)	4.9 (4.2)	5.0 (5.0)	5.1 (5.1)
R_{pim} (%)	4.1 (31.1)	3.8 (36.3)	3.7 (33.2)	3.6 (29.5)	3.8 (32.5)
I/σ(I)	9.3 (2.2)	8.5 (1.7)	8.5 (1.9)	9.1 (2.2)	8.6 (2.0)
Completeness (%)	99.5 (100)	99.2 (97.2)	99.4 (98.7)	99.5 (100)	99.5 (100)
Wilson B-factor (Å^2)	7.0	6.3	7.4	7.3	8.6
**Refinement**					
R_{work}/R_{free} (%)	11.9/15.5	12.4/15.0	12.3/15.5	12.2/15.6	12.3/16.5
RMSD Bond Length (Å)	0.02	0.02	0.02	0.02	0.02
RMDS Bond Angle (°)	2.1	2.4	2.5	2.5	2.5
ML Based ESU (Å)					
Av Protein B-factor (Å^2)	10.3	10.2	10.9	11.0	11.8
Av Water B-factor (Å^2)	26.6	24.5	30.5	28.0	31.2
**Ramachandran (%)**					
Favoured Regions	97.6	96.7	97.2	97.6	97.6
PDB Code	6ZU6	6ZUB	6ZUD	6ZUA	6ZUT

Table S2: Distances, bond angles and occupancies for key residues in the catalytic pocket.

**Residues/ ligands**	**Data1**	**Data2**	**Data3**	**Data4**	**Data5**
**Asp98**					
**Proximal**					
**Occupancy**	0.54	0.51	0.67	0.73	0.82
**Cγ-Oδ_1 (Å)**	1.25(8)	1.25(4)	1.25(3)	1.25(4)	1.25(3)
**Cγ-Oδ_2 (Å)**	1.26(5)	1.27(4)	1.28(3)	1.28(4)	1.30(3)
**Gate keeper**					
**Occupancy**	0.46	0.49	0.33	0.27	0.18
**Cγ-Oδ_1 (Å)**	1.24(5)	1.24(4)	1.25(7)	1.25(9)	1.25(11)
**Cγ-Oδ_2 (Å)**	1.27(5)	1.27(4)	1.26(7)	1.26(9)	1.26(12)
**His255**					
**Part 1**					
**Occupancy**	1.0		0.79	0.63	
**Cε_1-Nε_2-Cδ_2 (°)**	107(2)	107(2)	106(4)	107(4)	
**Cγ -Nδ_1-Cε_1 (°)**	110(2)	111(2)	114(6)	112(5)	
**Part 2**					
**Occupancy**			0.21	0.37	1.00
**Cε_1-Nε_2-Cδ_2 (°)**	-	-	109(10)	108(7)	107(3)
**Cγ -Nδ_1-Cε_1 (°)**	-	-	110(10)	110(10)	111(3)

**NO_2**					
**Occupancy**	0.75	0.68	0.55	0.40	
**B_{O1}-B_N-B_{O2} (Å^2)**	17-19-15	17-20-20	18-20-18	20-19-20	
**N-O_1 (Å)**	1.23(4)	1.24(5)	1.23(6)	1.24(9)	
**N-O_2 (Å)**	1.23(4)	1.24(5)	1.24 (7)	1.25(16)	
**O_1-N-O_2 (°)**	130(4)	138(5)	139 (6)	140(10)	
**NO**					
**Occupancy**				0.30	0.35
**B_O-B_N (Å^2)**				21-20	22-22
**N-O (Å)**	-	-		1.16(20)	1.15(20)
**W1**					
**Occupancy**		0.32	0.45	0.30	0.45
**B factor (Å^2)**		13.1	14.0	9.05	10.40
